# Epstein-Barr virus nuclear antigen 1 upregulates *Derlin1* and *PSMD10* expression in HeLa cells

**DOI:** 10.18632/genesandcancer.242

**Published:** 2025-08-06

**Authors:** Amir Hossein Alipour, Seyed Mohammad Ali Hashemi, Fatemeh Gharahkhani, Alireza Katanchi, Ali Farhadi, Jamal Sarvari

**Affiliations:** ^1^Department of Bacteriology and Virology, School of Medicine, Shiraz University of Medical Sciences, Shiraz, Iran; ^2^Department of Microbiology and Immunology, Faculty of Veterinary Medicine, University of Tehran, Tehran, Iran; ^3^Diagnostic Laboratory Sciences and Technology Research Center, School of Paramedical Sciences, Shiraz University of Medical Sciences, Shiraz, Iran; ^4^Gastroenterohepatology Research Center, Shiraz University of Medical Sciences, Shiraz, Iran; ^*^These authors contributed equally to this work

**Keywords:** cervical carcinoma, Epstein–Barr virus, EBNA1

## Abstract

Background: Epstein-Barr Virus (EBV), a potent viral carcinogen, plays a crucial role in the development of various malignancies. Among its proteins, EBV nuclear antigen-1 (EBNA1) stands out for its ability to modulate gene expression. In this study, we explored the impact of EBNA1 on the expression patterns of four cellular genes—Derlin1, ZEB1, CNN3, and PSMD10—in HeLa cells.

Materials and Methods: Three distinct categories of HeLa cells were established:

1. EBNA1-Transfected Cells: These cells were transfected with the EBNA1 gene.
2. Control Plasmid-Transfected Cells: These cells received transfection with a control plasmid.
3. Non-Transfected Cells (Control Group): These cells were not subjected to any transfection.

After RNA extraction, we employed real-time PCR to evaluate the transcriptional levels of four specific genes—Derlin 1, ZEB1, CNN3, and PSMD10—in each of the three cell groups. The Mann-Whitney *U*-test was subsequently utilized to compare means, and statistical significance was determined based on *p*-values below 0.05. Data were meticulously recorded in an Excel 2016 spreadsheet.

Results: The results demonstrated that HeLa cells transfected with the EBNA1 plasmid exhibited significantly increased expression levels of Derlin1 (*p* = 0.028) and PSMD10 (*p* = 0.028) genes compared to cells transfected with the control plasmid. However, the expression changes observed in CNN3 and ZEB1 were not statistically significant (*p* = 0.99 and *p* = 0.2, respectively).

Conclusions: Our findings suggest that increase expression levels of Derlin1 and PSMD10 genes in HeLa cells by the EBV-EBNA1 might induce cancer cell survival and accelerates the development of cervical cancer (CC). However, to establish a conclusive link between EBV-EBNA1 and CC progression, further investigations are warranted.

## INTRODUCTION

Cancer remains a global health challenge, and viruses are established contributors to many human malignancies [1]. Cervical carcinoma (CC) is one of the most prevalent cancers among women worldwide, accounting for approximately 1.6% of all female cancer-related deaths [2]. Human papillomaviruses (HPVs), particularly high-risk strains, are the primary etiological agents of CC, and their role in cervical tumorigenesis is well-documented [3]. However, HPV infection alone is insufficient for malignant transformation, and accumulating evidence suggests that additional cofactors and molecular events are required for cancer development and progression [4]. One such potential cofactor is Epstein–Barr virus (EBV), a double-stranded DNA virus with oncogenic potential in multiple epithelial and lymphoid malignancies, including nasopharyngeal carcinoma and gastric cancer [5]. Recent clinical and epidemiological studies have reported a significant presence of EBV in CC tissues, particularly in co-infection with high-risk HPV [6, 7]. EBV can infect cervical epithelial cells through C3d receptors on the uterine epithelium [8–10] and coinfection with HPV may synergistically enhance oncogenic transformation. Despite these findings, the molecular mechanisms underlying EBV’s contribution to CC pathogenesis remain largely unexplored. As a double-stranded DNA virus, EBV spans approximately 170 kb and is known to be carcinogenic [10]. Among EBV’s latent proteins, Epstein–Barr Virus Nuclear Antigen 1 (EBNA1) is of particular interest, as it is the only EBV protein consistently expressed in all latency types [11]. EBNA1 not only maintains the viral genome but also acts as a transcriptional regulator, capable of altering the expression of both viral and cellular genes [12–14]. Prior studies have implicated EBNA1 in promoting oncogenic pathways in other EBV-associated cancers [15], but its specific role in CC, particularly in the context of HPV-positive cells, has not been well characterized. To address this knowledge gap, we investigated whether EBNA1 expression affects the transcription of four cancer-related host genes in a CC model. These genes—Derlin1, ZEB1, CNN3, and PSMD10 (gankyrin)—were selected based on previous reports of their overexpression in CC and their established roles in cancer progression, metastasis, and immune regulation.

Derlin 1, a 22 kDa membrane protein located in the endoplasmic reticulum (ER), contains either four or six transmembrane domains. Its primary function is facilitating the translocation of unfolded or misfolded proteins from the ER lumen to the cytoplasm [16]. Notably, Derlin 1 is overexpressed in various cancer types, including cervical carcinoma, and its presence is closely associated with tumor development and progression [16, 17].

ZEB1, a transcription factor, is pivotal in regulating epithelial-mesenchymal transition (EMT) [18]. It selectively expresses in certain cancer cells, profoundly impacting their invasiveness and interactions within the tumor microenvironment [18]. Additionally, ZEB1 influences immune cell behavior during surveillance [19]. Notably, elevated ZEB1 expression correlates with unfavorable outcomes, including chemotherapy resistance [20].

The third gene assessed in our study was Calponin 3 (CNN3). CNN3, belonging to the calponin family of actin filament-associated proteins, is responsible for controlling actin cytoskeleton reorganization and dynamics [21, 22]. Furthermore, the overexpression of CNN3 in CC cells plays a role in stimulating the growth and metastasis of the cancer [23].

An enzyme called gankyrin, also referred to as 26S proteasome non-ATPase regulatory subunit 10, is generated in humans by the *PSMD10* gene [24]. Gankyrin is an oncoprotein that is a part of the proteasome’s 19S regulatory cap [24, 25]. Gankyrin also regulates the cell cycle by interacting with the cyclin-dependent kinases 4 (CDK4) and MDM2 which are both involved in tumor suppression and have been found mutated in many cancers [26]. Gankyrin has been demonstrated to be frequently overexpressed in cervical high-grade lesions and to be related to cervical carcinogenesis and metastasis [27]. In this regards, Hashemi et al. recently reported the increased expression of *PSMD10* in the MKN-45 cells following *EBNA1* transfection [28].

This study is among the first to directly assess the impact of EBV-EBNA1 expression on the transcriptional regulation of these genes in HPV-positive CC cells. Our findings may provide new insight into the cooperative oncogenic effects of EBV and HPV in CC and suggest potential molecular targets for further investigation.

## RESULTS

### Real-time PCR results of *Derlin1* gene expression following EBNA1 transfection

**Figure 1:**
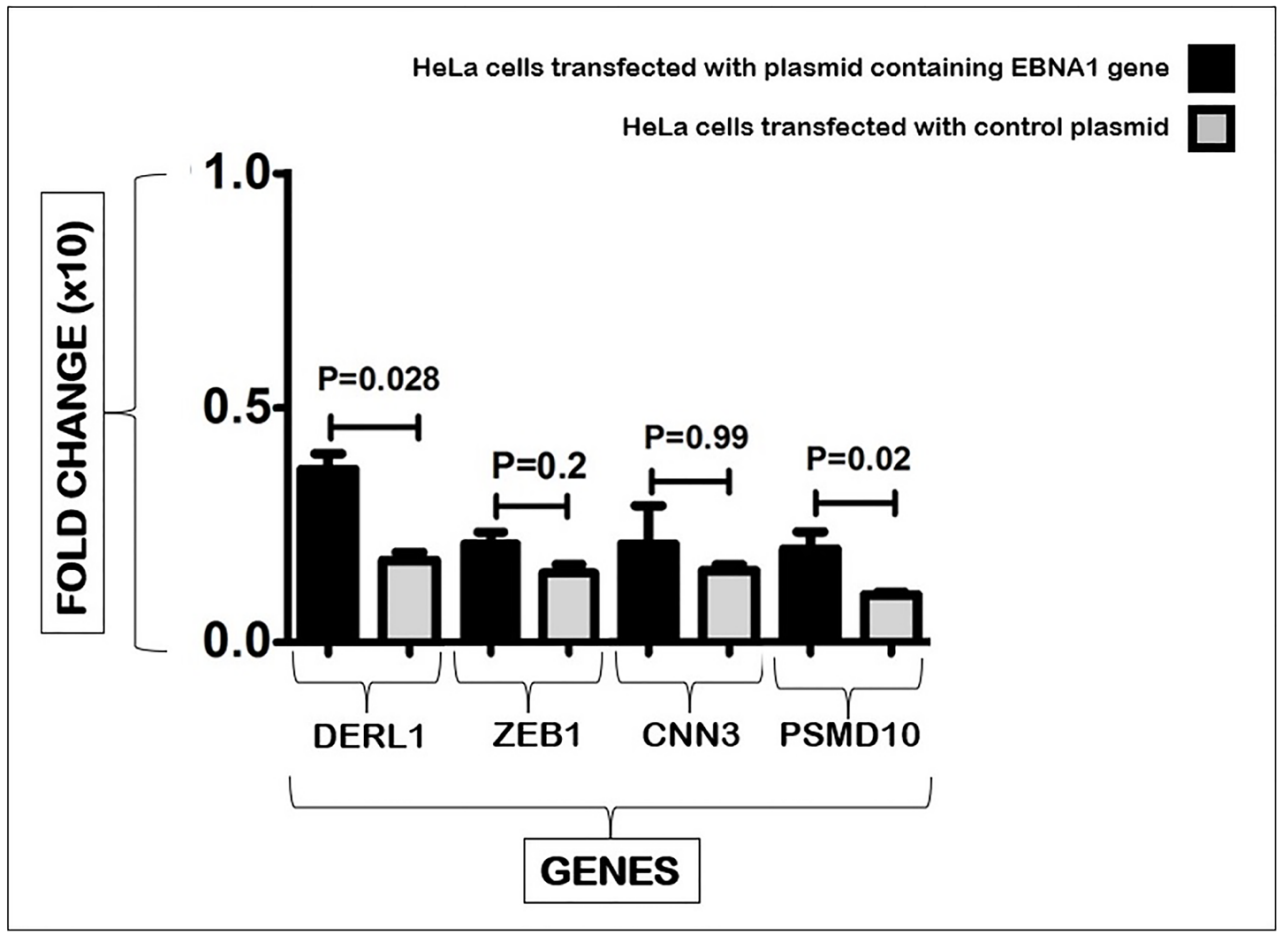
Real-time PCR analysis of *Derlin 1, ZEB1, CNN3 and PSMD10 genes* expression: *Derlin 1, ZEB1, CNN3 and PSMD10 genes* expression alterations in EBNA1 transfected HeLa cells versus mock plasmid-transfected cells.

*Derlin 1* cellular gene expression level was compared between EBNA1-transfected cells and controls (Figure 1). Real-time PCR results showed that the expression level of this gene was increased significantly about three-fold (*p* = 0.028) in the presence of EBNA1.

### Real-time PCR results of the *ZEB1* gene expression following EBNA1 transfection

In Figure 1, the expression of *ZEB1* gene was compared between EBNA1 transfected cells and control cells. Although EBNA1-transfected HeLa cells revealed an increase in the expression of this gene, this change was not significant (*p* = 0.2).

### Real-time PCR results of the *CNN3* gene expression following EBNA1 transfection

The *CNN3* gene expression was analyzed in EBNA1 transfected cells and control cells. While there was an observed increase in the expression of this gene in EBNA1-transfected HeLa cells, the difference was not statistically significant (*p* = 0.99).

### Real-time PCR results of *PSMD10* gene expression following EBNA1 transfection

Figure 1 shows the expression levels of the *PSMD10* gene in EBNA1-transfected cells and the cells transfected with mock plasmid. *PSMD10* gene was significantly more expressed in HeLa cells containing EBNA1 (*p* = 0.02) than in cells transfected with a mock plasmid; however, *PSMD10* gene expression was increased two-fold in cells harboring EBNA1.

## DISCUSSION

Scientists are actively investigating the connection between viruses and cancer. While it is well-established that EBV infection is linked to epithelial cancers such as gastric adenocarcinoma and nasopharyngeal carcinoma [5, 29, 30], its impact on other cancers like CC remains less understood. Recent clinical studies have detected EBV-DNA in cervical tissues from CC patients, raising questions about the virus’s potential role in disease progression [7, 31, 32]. Among EBV’s various proteins, EBNA1 stands out—it is present in all viral latency types and can influence gene expression by binding to their promoters [11, 12].

Our results from Real-time PCR demonstrated a significant three-fold rise in the expression level of *Derlin1* gene in the presence of EBNA1. In this regards, Li et al. conducted a study in which they observed a significant increase in the levels of *Derlin1* in CC tissues [33]. They also found a positive association between the expression of *Derlin1* and various clinical parameters such as tumor size, pathological grade, and lymph node metastasis in CC patients [33]. Furthermore, the researchers demonstrated that suppressing the expression of *Derlin1* in CC cell lines resulted in the inhibition of cell proliferation and migration [33]. Additionally, the knockdown of *Derlin1* induced apoptosis and influenced the expression of apoptosis-related proteins, including Bcl-2, Bax, Bim, caspase3, and caspase9 [33]. Fan et al. also discovered evidence indicating that DERL1 enhances tumor advancement through the AKT pathway, offering a novel potential target for the clinical management and diagnosis of hepatocellular carcinoma (HCC) [34]. Our findings suggest a potential association between EBNA1-induced Derlin1 expression and CC progression, which requires further validation.

According to our findings, the HeLa cell line expressing EBNA1 exhibits significantly higher *PSMD10* gene expression than the mock plasmid-transfected cells. PSMD10, commonly referred to as gankyrin, is a 26S proteasome regulatory member. Numerous investigations have revealed that gankyrin is overexpressed in a number of malignancies, which contributes to the growth of tumors and a poor prognosis [35, 36]. Consistent with our findings, Hashemi et al. conducted a study using MKN-45 cells transfected with the EBNA1 plasmid. They observed a substantial increase in *PSMD10* expression due to the presence of the EBNA1 protein [28]. Additionally, in a separate study, Hashemi and colleagues reported significant *PSMD10* overexpression in Burkitt lymphoma (BL) [37]. The 26S proteasome complex is required for ubiquitin-dependent protein degradation. Gankyrin regulates the tumor suppressors p53 and RB1 negatively. So the incorrect expression of this gene may contribute to cancer [38]. Gankyrin decreases apoptosis by destroying p53 and reducing the transcription of p53-dependent genes [35]. According to studies, gankyrin interacts with MDM2, enhancing the interaction between p53 and MDM2 which causes p53 to be ubiquitylated and subsequently destroyed through the proteasomal pathway [39]. These observations raise the possibility that EBNA1-induced Gankyrin expression could contribute to CC progression, though additional studies are needed to confirm this relationship and its therapeutic relevance [40].

According to the findings of the present study, the *ZEB1* gene exhibited higher expression in HeLa cells transfected with *EBNA1*; however, this change did not prove to be statistically significant. Farzanehpour and colleagues conducted a comparative analysis of the expression levels of some genes including *ZEB1* in HPV-induced CC [41]. They reported levels of *ZEB1* expression was increased in both cancerous and precancerous samples [41]. Ma et al., reported the increased expression of *ZEB1* has been associated with the irregular expression of E-cadherin, β-catenin, and N-cadherin, potentially contributing to the progression and metastasis of cervical squamous cell carcinoma [42]. Inhibiting the expression of *ZEB1* has the potential to hinder the transformation of invasive tumors into a mesenchymal phenotype in CC by decreasing the proliferation and mobility of CC cells. This indicates that targeting ZEB1 could serve as a promising therapeutic strategy for this type of cancer [43].

In the present study, the expression of the *CNN3* gene exhibited an increase in HeLa cells transfected with *EBNA1*. However, this difference in expression did not reach statistical significance. The presence of *CNN3* overexpression in CC tissues was reported, along with its involvement in enhancing proliferation, migration, and invasion in CC cells, and in speeding up the growth and metastasis of xenografted tumors in immunodeficient mice [23]. Additionally, the P1 subunit of the ribosomal protein lateral stalk, also referred to as RPLP1, is transcriptionally regulated by CNN3 [23]. This regulation plays a crucial role in the modulation of viability and motility of CC cells mediated by CNN3 [23]. In their study, Nair et al., have highlighted the involvement of CNN3 in lymph node metastasis and resistance to chemotherapy in colon cancer [44]. They propose that these CNN3-related actions are driven by significant oncogenic pathways [44]. Despite the fact that our findings indicated an overexpression of the CNN3 gene in the CC cell line containing EBNA1, the observed changes were not deemed statistically significant.

This study presents several limitations that should be acknowledged. First, experiments were performed using a single CC cell line (HeLa), which may not fully capture the biological diversity of HPV-positive and HPV-negative cervical cancers. Additionally, the absence of normal cervical epithelial cells as a control restricts our ability to evaluate gene expression changes against a physiological baseline. The analysis was limited to mRNA expression levels, without accompanying validation at the protein level using techniques such as Western blotting or immunohistochemistry. Moreover, no functional assays were conducted to explore the downstream biological effects of EBNA1-induced gene regulation—such as impacts on cell proliferation, migration, or apoptosis. Finally, as an *in vitro* study, these findings warrant further investigation through *in vivo* models to better understand the role of EBNA1 in CC progression.

Our findings suggest that EBNA1 may enhance Derlin1 expression in CC cells, potentially contributing to increased survival; however, further research is needed to confirm this hypothesis and assess its functional impact. The observed upregulation of PSMD10 in response to EBNA1 may play a role in CC biology, though additional functional and protein-level analyses are essential before clinical implications can be drawn. The results also indicated that *ZEB1*, and *CNN3* might not involved in the pathogenesis of CC infected with EBV. Further studies are recommended to clarify these results.

## MATERIALS AND METHODS

### Plasmids, bacterial transformation, and plasmid extraction

The pCEP4 plasmid (an EBV-based plasmid containing *EBNA1* and hygromycin B resistance genes) as well as a control plasmid that lacked *EBNA1* gene were used. The plasmids were transformed into *Escherichia coli* (*Top10* strain) and multiplied. To confirm the presence of the *EBNA1* gene in the plasmid, enzyme digestion and colony PCR methods were used. Then, these plasmids were extracted, and their quality and concentrations were determined using gel electrophoresis and spectrophotometry.

### Cell culture, transfection, and clonal selection by hygromycin B

HeLa cells (cervical adenocarcinoma cell line containing HPV-18) were cultured in RPMI-1640 containing 10% fetal bovine serum (FBS) at 37°C and 5% CO_2_ in a 6-well plate. After reaching a cell confluency of about 70%, a group of cells was transfected with *EBNA1* containing plasmid and the other group with the mock plasmid using an optimized concentration of DNA-fectamine (Bio Basic Inc., Canada). After 24 hours of transfection, cells were treated with 50 mg/ml hygromycin B to select cells with stable EBNA1 expression. These cells were cultured for 20 days in the presence of hygromycin B during several passages.

### Total RNA extraction, cDNA synthesis and validation of *EBNA1* gene expression

RNA Isolation Kit (Dena Zist, Mashhad, Iran) was used to extract the total RNA from HeLa cells. Electrophoresis and spectrophotometry were then applied to determine the quality and quantity of the isolated RNA, respectively. RNase-free DNase (Sinaclon, Tehran, Iran) was used to remove plasmid contamination from extracted RNA. An EasycDNA Synthesis Kit (AddScript RT-PCR SYBR Master, AddBio, Sweden) was used to reverse-transcribe 1000 ng/μl of extracted RNA from each sample into cDNA. Then, real-time PCR was applied to confirm EBNA1 expression.

### Quantitative reverse transcription PCR (qRT-PCR) assay

The primer sequences used for relative quantitative reverse transcription PCR (qRT-PCR) analysis of the target genes are detailed in Table 1. Gene expression levels of *Derlin1*, *ZEB1*, *CNN3*, and *PSMD10* were quantified using qRT-PCR on an ABI QuantStudio 3™ system (Applied Biosystems, Grand Island, NY, USA). The thermal cycling conditions included an initial denaturation at 95°C for 15 minutes, followed by 40 cycles of 15 seconds at 95°C and annealing/extension at 62°C. The β-actin gene served as the internal reference control for normalization [45].

**Table 1:** Primers used for evaluation of gene expression by relative quantitative real-time PCR

Gene name	Sequence	Product size	Primer position	References
***PSMD10***	5′-CTACTAGAACTGACCAGGACA-3′ 5′-GCCGCAATATGAAGAGGAG-3′	145 bp	F: 120–140 R: 246–264	[28]
***EBNA1***	5'-GGGTGGTTTGGAAAGCATCG-3' 5'-CTTACTACCTCCATATACGAACACA-3'	156 bp	F: 1257–1276 R: 1413–1387	[28]
***Derlin1***	5'-GAGAAGACAAGCAGCGGATG-3′ 5'-AAACACCCAGCAACAACCC-3′	171 bp	F:1488–1507 R:1640–1658	
***ZEB1***	5'-GCAGTCCAAGAACCACCCT-3′ 5'-ACACAAATCACAAGCATACATTCCA-3′	174 bp	F: 2831–2849 R: 2980–3004	
***CNN3***	5'-GAATGAGTGTGTATGGGCTTGG-3′ 5'-GTTCCTGTTCCTTGGCTTCC-3′	100 bp	F: 986–1007 R: 1066–1085	

### Statistical analysis

The biological experiments were performed in duplicate to ensure accuracy. To equalize the Ct values of the qRT-PCR runs, the CtNorm algorithm was utilized [46]. After Ct normalization, statistical analysis was conducted using the Mann-Whitney *U*-test in GraphPad Prism software, with statistical significance defined as a *P*-value < 0.05.
